# Effective Conversion of Amide to Carboxylic Acid on Polymers of Intrinsic Microporosity (PIM-1) with Nitrous Acid

**DOI:** 10.3390/membranes8020020

**Published:** 2018-04-18

**Authors:** Wei-Hsuan Wu, Paul Thomas, Paul Hume, Jianyong Jin

**Affiliations:** 1 School of Chemical Sciences, The University of Auckland, Auckland 1142, New Zealand; wwu182@aucklanduni.ac.nz; 2 School of Mathematical and Physical Sciences, University of Technology Sydney, Sydney, Broadway NSW 2007, Australia; paul.thomas@uts.edu.au

**Keywords:** polymers of intrinsic microporosity, gas separation membrane, carboxylated PIM-1

## Abstract

Carboxylate-functionalised polymers of intrinsic microporosity (C-PIMs) are highly desirable materials for membrane separation applications. The recently reported method to afford C-PIMs was via an extensive base hydrolysis process requiring 360 h. Herein, a novel and effective method to convert PIM-CONH_2_ to C-PIM using nitrous acid was studied. The chemical structure of C-PIM was characterised by ^1^H NMR, ^13^C NMR, FTIR, elemental analysis, UV-Vis, TGA and TGA-MS. Complete conversion from amide to carboxylic acid groups was confirmed. Decarboxylation of C-PIM was also successfully studied by TGA-MS for the first time, with a loss of *m*/*z* 44 amu (CO_2_) observed at the first degradation stage. TGA also revealed decreased thermal stability of C-PIM relative to PIM-CONH_2_ under both N_2_ and air atmosphere_._ Gel permeation chromatography (GPC) analysis showed continuous molecular weight degradation of C-PIM with extended reaction time. Aromatic nitration was also observed as a side reaction in some cases.

## 1. Introduction

The unprecedented energy consumption of the past century has led to dramatic increases in atmospheric CO_2_ concentration [1,2,3]. Because of this, there is a growing demand for efficient and cost-effective CO_2_ separation and capture technology [1,2,3]. In addition to the current chemical and thermal separation processes such as absorption, cryogenic distillation and pressure swing adsorption (PSA), membrane separation is slowly gaining ground in oil refineries and petrochemical plants due to its low energy consumption and environmental friendliness [4,5,6,7]. In the past three decades, polymeric membrane separations have exhibited strong growth rates and now have been installed for N_2_/O_2_ and CO_2_/CH_4_ separations plus H_2_ recovery from HCs, etc. Polymeric membranes enjoy low cost fabrication, easy scale-up, flexibility and mild operation conditions [8].

A unique class of microporous organic material (i.e., Polymers of Intrinsic Microporosity (PIMs)) was first developed by Budd and McKeown in 2004 [9]. Since the chemical structures and physical properties of the membrane materials influence their corresponding permeability and permselectivity, various new PIM structures and synthetics methods have been reported, in order to improve the gas separation performance [10,11,12,13,14,15]. The nitrile (–CN) group of PIM-1 is the most popular target for transformation, and has been converted to various CO_2_-philic functionalities such as nitrogen-containing groups [16,17,18,19,20,21,22] and carboxylic acid-based functional groups [23,24,25,26,27,28,29,30]. Carboxylate functionalised PIMs (C-PIMs) were inspired by the well-known carboxylic acid-containing polyimide based on the 6FDA-DABA, which exhibits superior gas separation performance after decarboxylation and/or crosslinking [31]. The first synthetic attempt toward C-PIM was reported in 2009 [24]. The synthesis was based on hydrolysis of the nitrile group under alkaline conditions. The resultant polymer showed an overall increase in permselectivity and decreased permeability [24]. However, in 2014, Satilmis et al. pointed out that the base-hydrolysed PIM-1 materials contained a mixture of amide, carboxylic acid, ammonium carboxylate and sodium carboxylate functional groups where the actual percentage of carboxylic acid was less than 20% [32]. Even after extensive base-hydrolysis, only 51% carboxylation was achieved [32]. In 2017, Santoso et al. reported a critical update on the characterisation of C-PIM [33]. Based on the spectroscopic data obtained from FT-IR, ^1^H NMR, ^13^C NMR, and ^1^H-^15^N HSQC, the amide and carboxylic acid groups were unambiguously distinguishable. The multiple proton signals between 7.0–8.5 ppm in ^1^H NMR were correctly assigned to the active amide protons (–C(=O)-NH_2_) whereas the carboxylic acid proton (–C(=O)-OH) is located further downfield in the range of 13.0–14.0 ppm. Moreover, the carbonyl stretch vibration at 1666 cm^−1^ observed by FTIR belongs to the amide carbonyl, whereas the carboxylic acid carbonyl stretch is located at 1720 cm^−1^ [33].

Most recently, Jeon et al. successfully obtained a fully converted C-PIM (>92 mol % COOH content) after an exhaustive 360 h (15 days) base-hydrolysis [34]. The resulting C-PIM film exhibited outstanding selectivity of 53.6 for CO_2_/N_2_ gas pair [34]. However, this extremely slow reaction rate is not ideal and could be a barrier to large scale industrial implementation. During the base hydrolysis process, the nitrile to amide conversion is relatively fast. The bottleneck appears to be the slow conversion from the amide to carboxylic acid [35]. The hydroxide (OH^−^) attacks the carbonyl carbon of the benzamide to give a tetrahedral intermediate. Since NH_2_^−^ is a stronger base and a less favourable leaving group, the tetrahedral intermediate can either revert to the amide starting material or form the carboxylic acid product.

Alternatively, conversion of nitrile to carboxylic acid via acidic hydrolysis route was published by Weng et al. in 2015. The reported reaction time was 48 h [23]. However, our group failed to reproduce the original experimental results.

A new synthesis of C-PIM via a fast and efficient reaction route is therefore required. As summarised in Table 1, herein we report an effective and fast method to convert amide-containing PIM-1 (i.e., PIM-CONH_2_) to C-PIM via reaction with nitrous acid. A combination of FTIR, ^1^H, ^13^C NMR, elemental analysis, UV-Vis, GPC, TGA, and TGA-MS were used to characterise the resulting C-PIM.

## 2. Materials and Methods

All chemicals and solvents were purchased from Sigma-Aldrich (St. Louis, MO, USA). 3,3,3′,3′-Tetramethyl-1,1′-spirobiindane-5,5′,6,6′-tetraol (Sigma-Aldrich, 96%) and 2,3,5,6-tetrafluoroterephthalonitrile (Sigma-Aldrich, 99%) were recrystallised before use and other chemicals and solvents were used as received without further treatment. However, caution is required: sodium nitrite needs to be handled with care due to high acute toxicity.

NMR spectra were recorded on Bruker DRX 400 MHz NMR spectrometers at 25 °C. The measurement frequencies for ^1^H NMR was 399.89 and 400.13 MHz. The chemical shifts for the NMR solvents reported for ^1^H and ^13^C in parts per million (ppm) are referenced to 7.26 ppm and 77.0 ppm for CDCl_3_, 2.5 ppm and 39.5 ppm for DMSO-*d*_6_, 1.7 ppm with 3.6 ppm and 67.2 ppm with 25.3 ppm for THF-*d*_8_, respectively. Broadband decoupled mode was used for recording ^13^C NMR spectra.

Infrared spectra were recorded on Nicolet iS50 FT-IR Spectrometer with a Universal ATR Sampling Accessory. Each sample was scanned 32 times at resolution of 4 cm^−1^.

UV-Vis spectra were recorded on a Shimadzu UV-2700 spectrophotometer. Pure solvents were used for baseline correction. For these measurements, PIM-CONH_2_ and C-PIM were dissolved in DMSO and THF at a concentration of 0.1 mg/mL.

Elemental analysis was performed in the Campbell Microanalytical Laboratory located at University of Otago. All measured microanalysis results have an uncertainty of ±0.4%.

Thermogravimetric analysis (TGA) was performed using a TG 5000 instrument. For polymer thermal degradation analysis, samples were heated from room temperature to 100 °C at a rate of 10 °C/min followed by isotherm for 60 min to remove water and solvents and then heated to 800 °C at a rate of 10 °C/min. Identical conditions were used for TGA measurements under nitrogen flow and air flow. For the thermal decarboxylation experiment, C-PIM was heated from room temperature to 100 °C at a rate of 10 °C/min followed by isotherm for 60 min and then heated to 350 °C at a rate of 10 °C/min followed by isotherm for 60 min. The leftover material was further characterised by FTIR.

Thermogravimetric analysis was coupled to a gas chromatography mass spectrometer (TG-GC-MS), which was used to analyse the gases evolved during pyrolysis. TG measurement was carried out using a Netzsch STA449 F5 Jupiter^®^ instrument. The experiments were carried out in a helium atmosphere flowing at 60 mL/min (40 mL/min directly to the furnace +20 mL to the furnace via the mass balance). High purity Helium 5.0 gas (99.999%) was supplied by Coregas. The sample insertion temperature was 40 °C followed by isotherm for 10 min. Samples were then heated to 1000 °C at a rate of 10 °C/min followed by isotherm for 20 min. Crucible cleaning was achieved by influx of 10 mL/min oxygen in the purge gas and cooling to 40 °C. The GC-MS configuration measurement range from 5 to 100 *m*/*z* was analysed using an Agilent 7890 GC with Agilent MSD 5977 EI.

PIM-1 is the polycondensation product of 3,3,3′,3′-Tetramethyl-1,1′-spirobiindane-5,5′,6,6′-tetraol and 2,3,5,6-tetrafluoroterephthalonitrile and was synthesised following the procedures reported by Budd and McKeown [9].

We previously reported the synthesis of PIM-CONH_2_ [36]. Typically, dimethyl sulfoxide (DMSO) (30.0 mL) was added to fine PIM-1 powder (0.500 g) and stirred at moderate speed for 1 h at R.T. Potassium carbonate (K_2_CO_3_) (0.500 g, 3.62 mmol) was then added to the stirring mixture to give a slightly basic pH 9–10 solution, followed by drop-wise addition of 25% hydrogen peroxide (H_2_O_2_) (5.0 mL, 32.64 mmol). The reaction was performed at RT for 24 h. Water (250 mL) was added at the end of the reaction and was further stirred rapidly overnight. A light-yellow polymer was collected by vacuum filtration and washed with water and methanol. The solid was dried in oven at 25 °C for 24 h to give PIM-CONH_2_ (0.48 g, yield > 80%). ^1^H NMR (400 MHz, DMSO-*d*_6_): *δ* ppm 7.94–7.57 (br, 2H), 6.80 (s, 2H), 6.18 (s, 2H), 2.21–2.07 (br, 4H), 1.29–1.21 (br, 12H). ^13^C NMR (100.57 MHz, DMSO-*d*_6_ ): *δ* ppm 162.0, 148.2, 145.5, 140.2, 140.0, 133.5, 115.7, 111.0, 110.0, 58.5, 56.7, 43.1, 31.1, 29.6.

Synthesis of C-PIM using nitrous acid: In a pressure tube, PIM-CONH_2_ (50 mg) was suspended in a stirred mixture of MeCN (0.8 mL) and 2M aqueous H_2_SO_4_ (0.6 mL, 1.2 mmol). NaNO_2_ (0.150 g, 2.4 mmol) was added portion-wise at 0 °C. After addition, the cap was screwed tightly, and the reaction vessel immersed in oil at 90 °C for 6 h. The reaction mixture was then diluted with water (4 mL) and extracted with ethyl acetate (8 mL). The organic layer was then concentrated in vacuo. The crude product was then dissolved in THF, filtered and solution concentrated in vacuo and dried in a vacuum oven at 70 °C overnight. The crude polymer was re-dissolved in THF, precipitated by addition of MeOH and then dried in vacuum to give C-PIM (35 mg, yield = 70%) as a dark yellow powder which was fluorescent under UV. ^1^H NMR (400 MHz, DMSO-*d*_6_): *δ* ppm 13.84 (br, 2H), 6.87 (s, 2H), 6.29 (s, 2H), 2.26–2.11 (br, 4H), 1.33–1.25 (br, 12H). ^13^C NMR (100.57 MHz, DMSO-*d*_6_ ): *δ* ppm 162.3, 148.6, 145.7, 139.8, 133.6, 113.4, 111.3, 110.1, 58.3, 56.7, 43.1, 31.0, 29.5.

## 3. Results

**Scheme 1:**
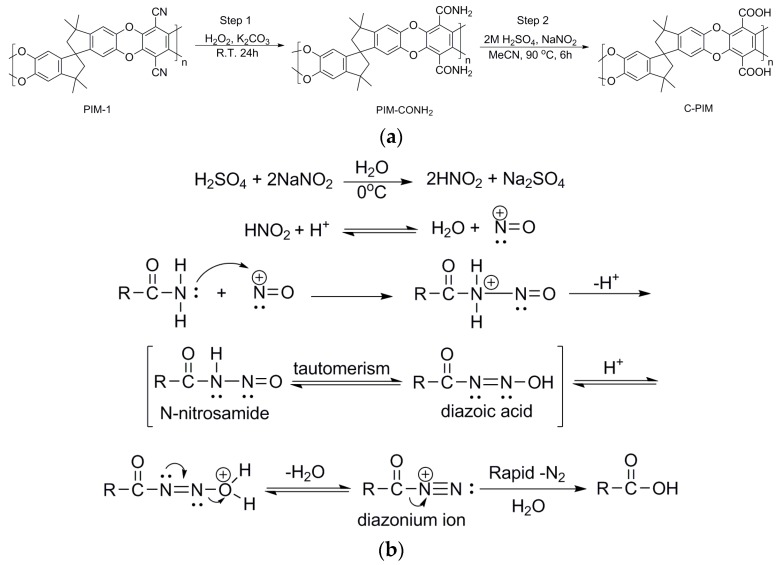
(**a**) Two-step synthesis of C-PIM via PIM-CONH_2_. (**b**) Proposed reaction mechanism for the conversion of amides to carboxylic acid using nitrous acid [38].

Previously reported post-polymerisation modifications of PIM-1 to C-PIM have utilised either acid or base-catalysed hydrolysis procedures [23,24,32,33,34,37]. In both pathways, PIM-CONH_2_ is formed as an intermediate. This most likely results in the co-existence of two distinct functional groups (amide and carboxylic acid) on a single polymer chain. Furthermore, when using the basic conditions reported by Jeon et al. the C-PIM is obtained after an extremely long reaction time (15 days) [34]. Based on these issues, we have developed a two-step approach to C-PIM as shown in Scheme 1a. PIM-1 is first converted to PIM-CONH_2_ using hydrogen peroxide. Pure PIM-CONH_2_ is purified and isolated. In the second step, PIM-CONH_2_ undergoes reaction with nitrous acid (HNO_2_) to afford C-PIM. Since nitrous acid is unstable, it is prepared in situ by the reaction of sodium nitrite and dilute acid in the absence of heat. We have previously reported the detailed synthesis of PIM-CONH_2_ [36]. Here we only focus on the second reaction. Optimisation of reaction solvent, the choice of the type of acid and acidity, and reaction time are discussed in detail below.

Pioneered by George Olah et al., nitrous acid has found use as an alternative method to convert amide compounds that are resistant to standard hydrolysis to the corresponding carboxylic acids [38]. In 1965, Olah et al. reported the conversion sterically hindered benzamides and benzenesulfonamides to the corresponding acids by treatment with nitrosonium tetrafluoroborate at 50 °C in acetonitrile, in high yield (>80%) [38]. The amide can react with the nitrous acid to give the corresponding carboxylic acid and nitrogen gas. Scheme 1b gives the proposed reaction mechanism. The key reactive species is the highly electrophilic nitrosonium (NO^+^) cation, which is readily added to amide nitrogen, resulting in the formation of the corresponding *N*-nitrosamide. The *N*-nitrosamide tautomerises to the diazoic acid which forms a diazonium cation by losing a molecule of water. The diazonium cation decomposes rapidly through evolution of nitrogen and forms acyl carbocation. Finally, the acyl carbocation is quenched by water to give the carboxylic acid product.

**Figure 1:**
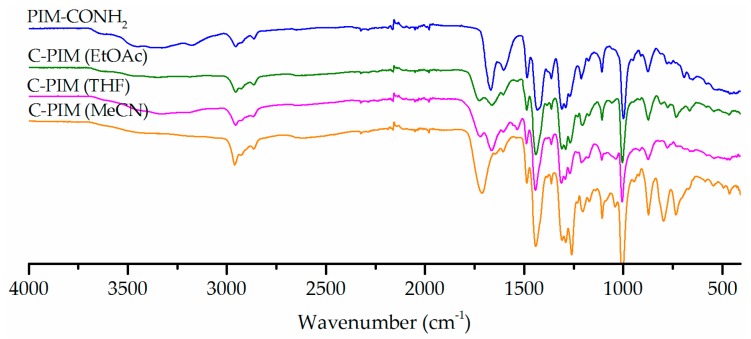
FTIR spectra of the reaction products in three solvents (EtOAc, THF and MeCN) with NaNO_2_ and 2M H_2_SO_4_ for 6 h.

First, three common organic solvents: ethyl acetate (EtOAc), tetrahydrofuran (THF) and acetonitrile (MeCN) were tested as reaction media. The reaction temperature was adjusted with the solvent’s boiling point. Therefore, the temperature was set at 70 °C for THF, 80 °C for EtOAc and 90 °C for MeCN. At the beginning of the reaction, PIM-CONH_2_ was not soluble in any of these solvents. As the reaction proceeded, the reaction mixtures became partially soluble in EtOAc and MeCN and fully soluble in THF. Figure 1 shows the effect of solvent on the reaction of PIM-CONH_2_ with NaNO_2_ and 2M H_2_SO_4_ for 6 h. Conversion was observed in all three solvents as assessed by FTIR which exhibited a carboxylic acid carbonyl stretching peak at 1720 cm^−1^. However, the experiments carried out in EtOAc and THF still showed the peak at 1666 cm^−1^ which corresponds to the stretch vibration of amide carbonyl. Furthermore, a new peak at 1545 cm^−1^ was present in these two solvent systems. In prior literature, this type of signal was found in nitrated polyphenylene oxide (PPO-NO_2_) reported by Bhole et al. [39] and nitrated poly (diphenylacetylene) reported by Sakaguchi et al. [40]. Their FTIR showed absorption peaks at 1532 cm^−1^ and 1364 cm^−1^, which corresponded to the aromatic –NO_2_ antisymmetric stretch and –NO_2_ symmetrical stretch, respectively. This is therefore strong evidence that nitration of PIM-CONH_2_ has also occurred in EtOAc and THF. On the basis of these results, MeCN is best suited for the present reaction, in which the product gave the most intense carboxylic acid carbonyl stretching peak, with minimal amide carbonyl stretching. Notably no nitration side reaction occurred in MeCN.

**Figure 2:**
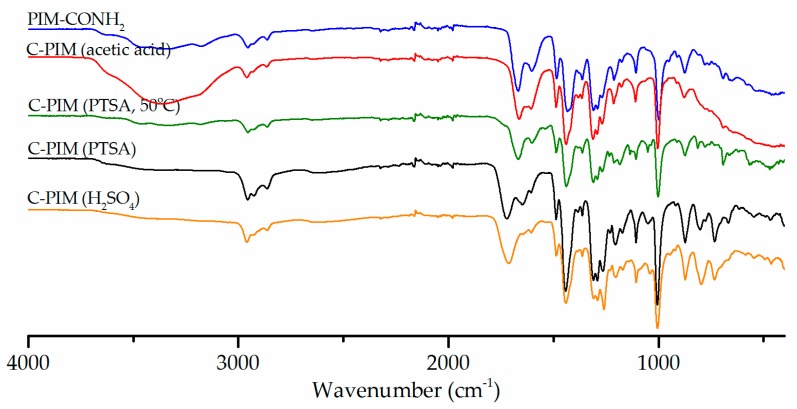
FTIR spectra comparison of the reaction products in three acid systems (acetic acid, PTSA, H_2_SO_4_) with NaNO_2_ in MeCN at 90 °C for 6 h.

Secondly, besides the combination of sodium nitrite with sulfuric acid, organic acids such as acetic acid and *p*-toluenesulfonic acid (PTSA) with sodium nitrite [41,42,43] were tested. The use of organic acids was expected to be more compatible with reaction solvent (MeCN) while still facilitating the generation of nitrous acid. Figure 2 shows the effect of the acid on the reaction outcome using NaNO_2_ in MeCN. All acids were made into the same concentration of 2M and performed under otherwise identical reaction conditions (90 °C for 6 h). A lower temperature reaction at 50 °C was also performed for PTSA in accordance with literature precedent [43]. The acetic acid system showed no conversion, as the parent amide absorption peak at 1666 cm^−1^ was still intact and no carboxylic acid carbonyl absorption peak at 1720 cm^−1^ was observed. This result was not surprising since when H_2_SO_4_ and PTSA were mixed with sodium nitrite, nitrogen gas was evolved which confirmed that nitrous acid had been produced, but this was not the case with acetic acid. This is likely due to the lower strength of acetic acid compared to the other acids. The temperature dependence of the reaction was also observed in the reaction performed using PTSA. The reaction with PTSA at 50 °C is not as effective as the one at 90 °C. At 50 °C, the amide stretch at 1666 cm^−1^ was still the dominant absorption in the IR spectrum, with a very weak carboxylic acid carbonyl absorption peak at 1720 cm^−1^. Comparing H_2_SO_4_ and PTSA, the former resulted in a higher conversion rate.

**Figure 3:**
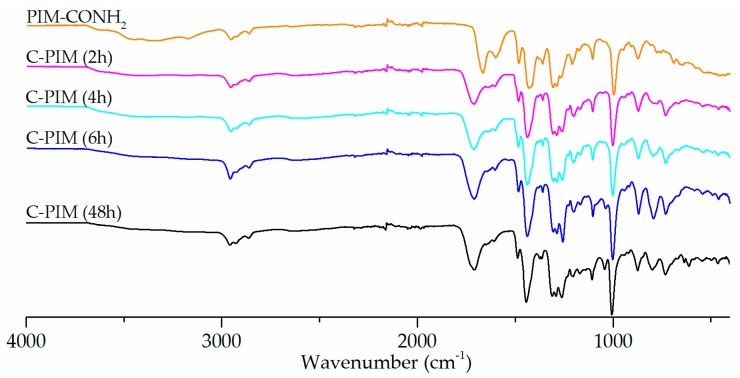
FTIR spectra of reaction of PIM-CONH_2_ with NaNO_2_ and H_2_SO_4_ in MeCN at 90 °C at reaction times from 2–48 h.

After identifying the best reaction solvent and acid, the reaction kinetics were monitored by FTIR. Figure 3 shows the reaction progression between 2 to 48 h. Remarkably, under the optimised reaction conditions (MeCN, NaNO_2_ and H_2_SO_4_ at 90 °C) the conversion from amide to carboxylic acid was pleasingly fast. Within 2 h, the amide band at 1666 cm^−1^ had nearly disappeared and was taken over by the carboxylic acid peak at 1720 cm^−1^. There was no obvious change in the intensity of the C=O band of carboxylic acid after 6 h. Clearly, the reaction kinetics for the nitrous acid reaction are much faster than acid or base catalysed hydrolysis.

**Figure 4:**
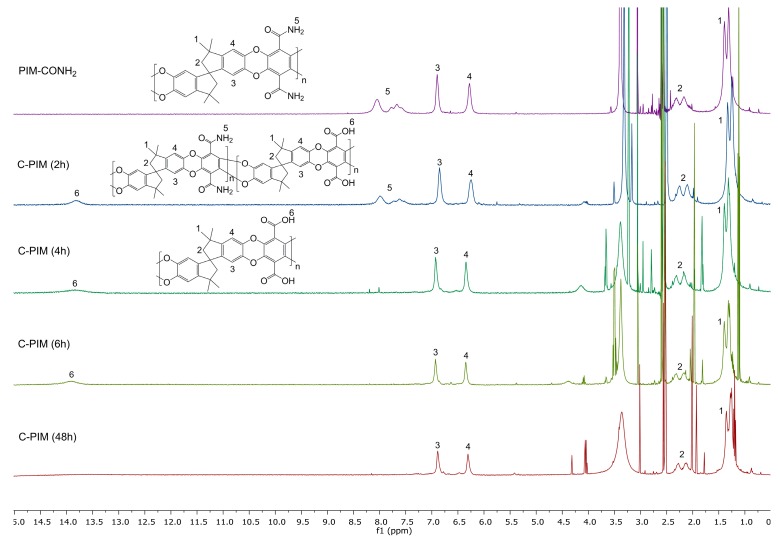
^1^H NMR spectra of C-PIM at reaction times from 2 h to 48 h.

The ^1^H NMR spectra were also used to probe the reaction kinetics. Figure 4 compares the ^1^H NMR spectra of PIM-CONH_2_, C-PIM (2 h), C-PIM (4 h), C-PIM (6 h) and C-PIM (48 h) samples. For PIM-CONH_2_, multiple peaks are observed at 7.0–8.5 ppm which are due to the four active amide (–C(=O)NH_2_) protons. For the C-PIM (2 h) sample, both amide and carboxylic acid (–C(=O)OH) proton resonances in the 13.0–14.0 ppm region were present. As the reaction progressed to 4 h, the amide proton region (7.0–8.5 ppm) turned to flat baseline. After 6 h reaction time, there were no obvious changes in ^1^H NMR spectra, consistent with the FTIR results. We therefore concluded that the conversion was complete after 6 h.

The conversion of the amide to the carboxylic acid was also verified by elemental analysis (Table 2). Increased percentages of hydrogen atoms (4.47% to 5.33%) and oxygen atoms (14.69% to 23.90%) were observed from the conversion of PIM-1 to PIM-CONH_2_, where four additional hydrogen atoms and two additional oxygen atoms were gained per repeat unit. From PIM-CONH_2_ to C-PIM, the percentage of hydrogen atoms was decreased (5.33% to 4.80%) with an increase in oxygen atoms (23.90% to 30.74%), where two hydrogen atoms were lost and two more oxygen atoms were gained for each repeat unit in C-PIM polymer. Most significantly, the decrease of nitrogen content from 5.24% in PIM-CONH_2_ to 0.91% in C-PIM indicated successful conversion of the amide groups to carboxylic acid functionalities.

The solubility of C-PIM in common organic solvents is very important for applications involving membrane casting. In Table 3, the solubility of PIM-1, PIM-CONH_2,_ and C-PIM are compared. It was well known that PIM-1 was readily soluble in tetrahydrofuran (THF), dichloromethane (DCM), and chloroform (CHCl_3_), but insoluble in polar aprotic solvents such as dimethyl formamide (DMF), dimethyl acetamide (DMAc), and *N*-methyl pyrrolidone (NMP). In contrast to PIM-1, PIM-CONH_2_ showed good solubility in polar aprotic solvents such as DMF, DMAc, NMP, and DMSO. The C-PIM prepared in this work exhibited wide solubility in many common solvents except less polar ones such as DCM and CHCl_3_. Interestingly, C-PIM was even soluble in acetone. The higher solubility of C-PIM than PIM-CONH_2_ was attributed to favourable interactions between the carboxylic acid groups of C-PIM with the solvent molecules.

**Figure 5:**
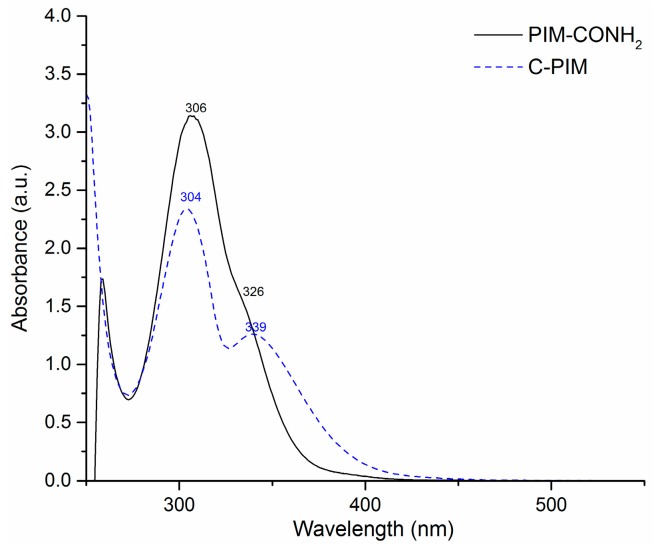
UV-Vis spectra of PIM-CONH_2_ in DMSO and C-PIM in THF.

The UV-Vis spectra of PIM-CONH_2_ and C-PIM with same concentration (0.1 mg/mL) are shown in Figure 5. For the starting polymer PIM-CONH_2_, the absorption maximum (λ_max_) was at 306 nm corresponding to the π-π* transition while the shoulder peak at 326 nm corresponds to the n-π* transition of the carbonyl group. For the product polymer C-PIM, λ_max_ at 304 nm corresponds to the π-π* transition and λ_max_ at 339 nm arise from the n to π* transition. A red shift of about 13 nm was observed for the n-π* transition (–C=O) of C-PIM compared to that of PIM-CONH_2_. This indicated that the energy gap between n orbital and π* orbital for C-PIM was smaller than that of PIM-CONH_2_.

**Figure 6:**
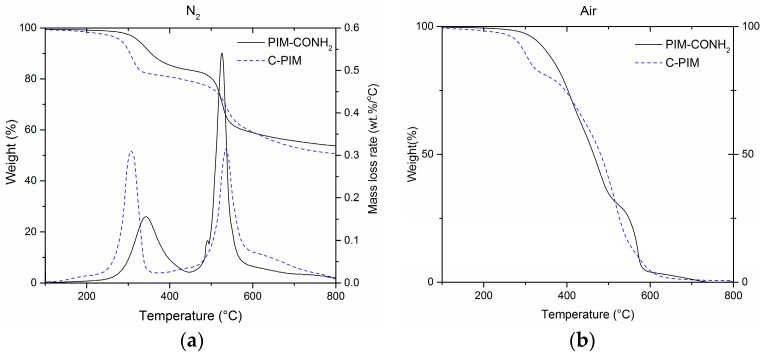
(**a**) Thermogravimetric analysis of PIM-CONH_2_ and C-PIM at 10 °C/min under N_2_ atmosphere. The left axis marks weight loss change in % and the right axis marks mass loss rate in wt. %/°C; (**b**) Thermogravimetric analysis of PIM-CONH_2_ and C-PIM at 10 °C/min under air atmosphere.

The thermal stability of C-PIM and PIM-CONH_2_ were investigated by TGA under nitrogen and air, respectively. Figure 6a shows the pyrolysis behaviour of PIM-CONH_2_ and C-PIM under a nitrogen atmosphere. Both C-PIM and PIM-CONH_2_ clearly showed two distinct stages of thermal degradation. Subsequently, two main peaks can be clearly resolved from the differential TGA curve (DTG). In PIM-CONH_2_, the first degradation stage is associated with a mass loss of ~15% in the range of 270–450 °C. This was attributed to the release of the labile pendant group (i.e., amide). During the second degradation stage, a mass loss of ~25% was observed in the range of 450–600 °C which was ascribed to decomposition of the PIM backbone. The C-PIM gave a similar TGA thermogram with two degradation stages. However, the conversion of the amide group to the carboxylic acid group decreased in thermal stability. Under nitrogen, the onset temperature for decarboxylation was measured to be 250 °C, with a major loss of CO_2_ at 307 °C. A total mass loss of 16% was observed in the range of 250–370 °C corresponding to the decarboxylation. As the theoretical value for full removal of the side chain is 18%, this means that near-total decarboxylation had occurred. This decarboxylation result is in agreement with the decarboxylation of polythiophene ester reported by Søndergaard et al. [44]. The second degradation stage of C-PIM occurred between 450–600 °C, and was associated with a mass loss of 20%.

Moreover, as analysed from the DTG curves, in the first stage of degradation (loss of pendant group), the maximum mass loss rate (r_max_) of the C-PIM was r_max_ = 0.31 %/°C at 307 °C which was twice the value of PIM-CONH_2_ with r_max_ = 0.16 %/°C at 342 °C. This suggests that the rate of decarboxylation is faster than the de-amidation rate. In the second stage of degradation (main chain breakdown), C-PIM has r_max_ = 0.32 %/°C at 536 °C while PIM-CONH_2_ has a very large r_max_ = 0.54 %/°C at 526 °C. This interesting DTG data might reveal that the C-PIM polymer after decarboxylation stage is relatively more stable as a result of decarboxylation-induced aryl radical homo-coupling (i.e., cross-linking).

Figure 6b shows the pyrolysis behaviour of PIM-CONH_2_ and C-PIM under an air atmosphere. The C-PIM still showed decarboxylation in the range of 270–350 °C. This indicates that decarboxylation of C-PIM was not affected by the oxidative environment. For PIM-CONH_2_, there was no obvious first stage degradation. Both C-PIM and PIM-CONH_2_ decomposed completely as 100% weight loss was observed at the end of pyrolysis at 800 °C, whereas under the N_2_ atmosphere, ~50% char yields were obtained.

**Figure 7:**
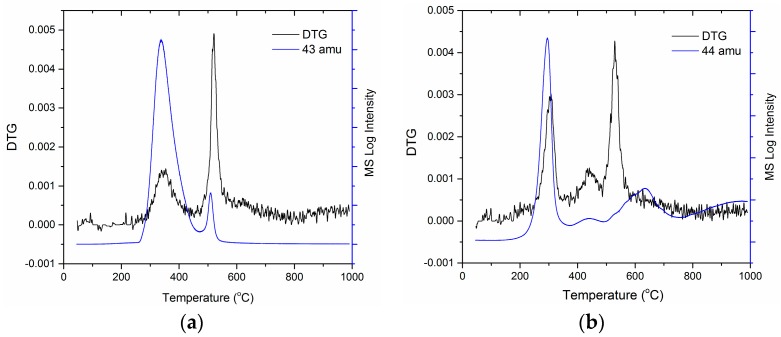
Thermogravimetric analysis combined with mass spectroscopy (TGA-MS). (**a**) DTG curve of PIM-CONH_2_ overlaid with MS trace of *m*/*z* 43 amu and (**b**) DTG curve of C-PIM overlaid with MS trace of *m*/*z* 44 amu.

For the first time, TGA-MS was used to analyse the composition of the evolved gases from C-PIM and PIM-CONH_2_ during TGA scans, focusing on their first degradation stages (decarboxylation and de-amidation, respectively). In Figure 7a, the DTG curve of PIM-CONH_2_ is overlaid with MS signal for the molecular ion with *m*/*z* 43 amu evolved during the TGA scan. A mass loss of 43 amu (CONH) showed the best match with the PIM-CONH_2_ first DTG peak at 350 °C. This confirmed that the first mass loss peak resulted from release of the amide group. In Figure 7b, the DTG curve of C-PIM is overlaid with the MS signal for the molecular ion of *m*/*z* 44 amu evolved during the TGA scan. The MS peak associated with the molecular weight of 44 (CO_2_) showed the perfect match with the C-PIM first DTG peak at 300 °C, which confirms that the first mass loss peak is due to the decarboxylation of C-PIM.

**Figure 8:**
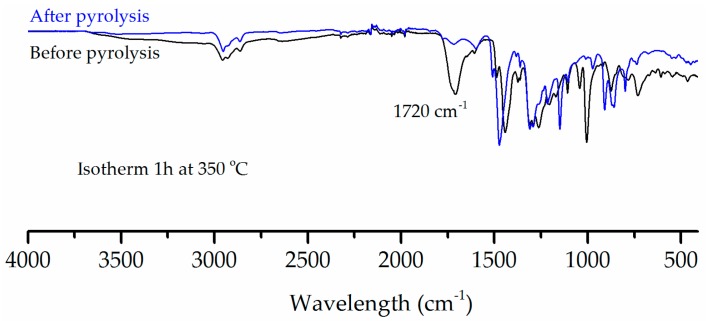
FTIR spectra comparison of the C-PIM samples before and after decarboxylation via isothermal pyrolysis at 350 °C for 60 min.

Figure 8 compares the FTIR of C-PIM samples before and after pyrolysis. The pyrolysed C-PIM sample was held isothermally at 350 °C for 60 min under nitrogen. The pyrolysis residue clearly shows the disappearance of the original carboxylic acid C=O stretch at 1720 cm^−1^. This further confirmed that the first degradation stage is the decarboxylation of C-PIM.

## 4. Discussion

**Figure 9:**
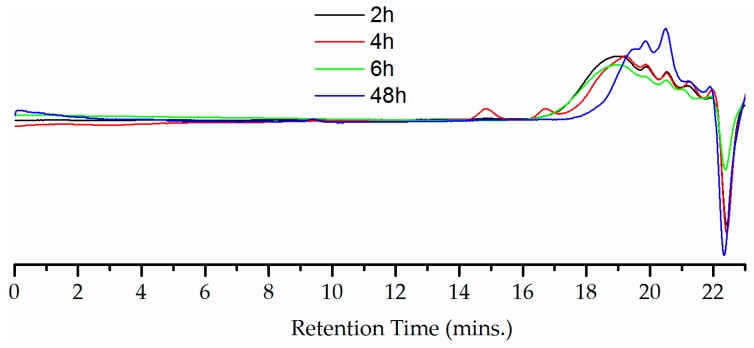
GPC chromatograph showing molecular weight change of C-PIM at different reaction times from 2 h to 48 h using THF as eluent.

The film forming ability and mechanical properties of PIMs are important for membrane applications. The C-PIM produced via acid hydrolysis reported by Weng et al. [23] and the base hydrolysis reported by Jeon et al. [34] had the ability to form free standing isotropic films. Film casting of C-PIM from this work was attempted. But unfortunately, the C-PIM produced via nitrous acid route was unable to form a flexible film. It appears likely that the main reason for this was that the starting material PIM-CONH_2_ was unable to form a film, so the C-PIM carried on this disadvantage. Moreover, the GPC analysis of C-PIM in THF (Figure 9) revealed that molecular weight degradation had occurred during the nitrous acid reaction. It was observed that longer reaction times, were associated with decreases in molecular weight.

In our approach, another side reaction is the aromatic ring nitration. In the early discussion of the reaction solvent choice, aromatic nitration was observed for the reactions conducted in both THF and EtOAc. Aromatic nitration is caused by the nitronium ion (NO_2_^+^) in the reaction system. A typical example of nitration of polystyrene is reported by Philippides et al. where they used nitric acid and sulfuric acid mixture [45]. In the nitrous acid reaction, it seems that nitric acid (HNO_3_) was also inevitably produced, which in turn resulted in the formation of NO_2_^+^.

## 5. Conclusions

A novel and effective method to convert PIM-1 to C-PIM was studied. The C-PIM was successfully prepared from PIM-CONH_2_ using nitrous acid as a key reagent. The optimum reaction conditions were NaNO_2_, H_2_SO_4_ in MeCN at 90 °C for 6 h. The resulting C-PIM was soluble in various organic solvents such as THF, acetone, DMSO and NMP. The chemical structure of C-PIM was characterised by ^1^H NMR, ^13^C NMR, FTIR, elemental analysis, UV-Vis, TGA and TGA-MS. These measurements confirmed complete conversion of the amide groups to carboxylic acids. Thermal analysis by TGA showed that C-PIM has two degradation stages where the first stage was decarboxylation and second stage was backbone decomposition. For the first time, the decarboxylation product was confirmed by TGA-MS, in which a loss of *m*/*z* 44 amu (CO_2_) at the first degradation stage was found. TGA also showed that the thermal stability of C-PIM was less than PIM-CONH_2_ under both N_2_ and air atmosphere. GPC analysis showed continuous molecular weight degradation of C-PIM with extended reaction times. Aromatic nitration was observed as a side-reaction in EtOAc and THF. Further optimisation of this reaction is currently underway.

## Figures and Tables

**Table 1:** Summary of C-PIM synthesis methods.

Methods	Base Hydrolysis	Acid Hydrolysis	This Work
Starting Material	PIM-1	PIM-1	PIM-CONH_2_
Reaction Conditions	20% NaOH at 120 °C, 5 h	H_2_SO_4_, H_2_O and Acetic acid at 105 °C, 48 h	NaNO_2_, H_2_SO_4_ and MeCN at 90 °C, 6 h

**Table 2:** Elemental analysis results for PIM-1, PIM-CONH_2_, and C-PIM.

Samples	C%	H%	N%	O% *
PIM-1	74.65	4.47	6.19	14.69
PIM-CONH_2_	65.53	5.33	5.24	23.90
C-PIM	63.55	4.80	0.91	30.74

* O% = 100–(C% + H% + N%).

**Table 3:** Solubility comparison of PIM-1, PIM-CONH_2_ and C-PIM in organic solvents.

Solvent	PIM-1	PIM-CONH_2_	C-PIM Obtained in This Work
DCM	soluble	insoluble	insoluble
EtOAc	insoluble	insoluble	partially soluble
CHCl_3_	soluble	insoluble	insoluble
THF	soluble	partially soluble	soluble
DMF	insoluble	soluble	soluble
DMAc	insoluble	soluble	soluble
NMP	insoluble	soluble	soluble
MeOH	insoluble	partially soluble	partially soluble
MeCN	insoluble	insoluble	partially soluble
EtOH	insoluble	insoluble	partially soluble
Acetone	insoluble	insoluble	soluble
DMSO	insoluble	soluble	soluble

## References

[1] Pachauri R.K., Allen M.R., Barros V.R., Broome J., Cramer W., Christ R., Church J.A., Clarke L., Dahe Q., Dasgupta P. (2014). Climate Change 2014: Synthesis Report. Contribution of Working Groups I, II and III to the Fifth Assessment Report of the Intergovernmental Panel on Climate Change.

[2] Sokolov A.P., Stone P.H., Forest C.E., Prinn R., Sarofim M.C., Webster M., Paltsev S., Schlosser C.A., Kicklighter D., Dutkiewicz S. (2009). Probabilistic forecast for twenty-first-century climate based on uncertainties in emissions (without policy) and climate parameters. J. Clim..

[3] Agiaye E.O., Othman M. (2015). CO_2_ Capture & Utilization: Harnessing the CO_2_ Content in Natural Gas for Environmental and Economic Gains. Proceedings of the SPE Nigeria Annual International Conference and Exhibition.

[4] Chakravarti S., Gupta A., Hunek B. (2001). Advanced Technology for the Capture of Carbon Dioxide from Flue Gases. Proceedings of the First National Conference on Carbon Sequestration.

[5] Pires J., Martins F., Alvim-Ferraz M., Simões M. (2011). Recent developments on carbon capture and storage: An overview. Chem. Eng. Res. Des..

[6] Riemer P.W. (1993). The Capture of Carbon Dioxide from Fossil Fuel Fired Power Stations.

[7] Pfaff I., Kather A. (2009). Comparative thermodynamic analysis and integration issues of CCS steam power plants based on oxy-combustion with cryogenic or membrane based air separation. Energy Procedia.

[8] Scholes C.A., Kentish S.E., Stevens G.W. (2009). Effects of minor components in carbon dioxide capture using polymeric gas separation membranes. Sep. Purif. Rev..

[9] Budd P.M., Ghanem B.S., Makhseed S., McKeown N.B., Msayib K.J., Tattershall C.E. (2004). Polymers of intrinsic microporosity (PIMs): Robust, solution-processable, organic nanoporous materials. Chem. Commun..

[10] Zhang J., Jin J., Cooney R., Fu Q., Qiao G.G., Thomas S., Merkel T.C. (2015). Synthesis of perfectly alternating copolymers for polymers of intrinsic microporosity. Polym. Chem..

[11] Zhang J., Jin J., Cooney R., Zhang S. (2015). Synthesis of polymers of intrinsic microporosity using an ab-type monomer. Polymer.

[12] Zhang J., Jin J., Cooney R., Zhang S. (2015). Fluoride-mediated polycondensation for the synthesis of polymers of intrinsic microporosity. Polymer.

[13] Zhang J., Kang H., Martin J., Zhang S., Thomas S., Merkel T.C., Jin J. (2016). The enhancement of chain rigidity and gas transport performance of polymers of intrinsic microporosity via intramolecular locking of the spiro-carbon. Chem. Commun..

[14] Tiwari R.R., Jin J., Freeman B.D., Paul D.R. (2017). Physical aging, CO_2_ sorption and plasticization in thin films of polymer with intrinsic microporosity (PIM-1). J. Membr. Sci..

[15] Starannikova L., Belov N., Shantarovich V., Zhang J., Jin J., Yampolskii Y. (2018). Effective increase in permeability and free volume of PIM copolymers containing ethanoanthracene unit and comparison between the alternating and random copolymers. J. Membr. Sci..

[16] Du N., Park H.B., Robertson G.P., Dal-Cin M.M., Visser T., Scoles L., Guiver M.D. (2011). Polymer nanosieve membranes for CO_2_-capture applications. Nat. Mater..

[17] Du N., Robertson G.P., Dal-Cin M.M., Scoles L., Guiver M.D. (2012). Polymers of intrinsic microporosity (PIMs) substituted with methyl tetrazole. Polymer.

[18] Patel H.A., Yavuz C.T. (2012). Noninvasive functionalization of polymers of intrinsic microporosity for enhanced CO_2_ capture. Chem. Commun..

[19] Swaidan R., Ghanem B.S., Litwiller E., Pinnau I. (2014). Pure-and mixed-gas CO_2_/CH_4_ separation properties of PIM-1 and an amidoxime-functionalized PIM-1. J. Membr. Sci..

[20] Mason C.R., Maynard-Atem L., Al-Harbi N.M., Budd P.M., Bernardo P., Bazzarelli F., Clarizia G., Jansen J.C. (2011). Polymer of intrinsic microporosity incorporating thioamide functionality: Preparation and gas transport properties. Macromolecules.

[21] Mason C.R., Maynard-Atem L., Heard K.W., Satilmis B., Budd P.M., Friess K., Lanč M., Bernardo P., Clarizia G., Jansen J.C. (2014). Enhancement of CO_2_ affinity in a polymer of intrinsic microporosity by amine modification. Macromolecules.

[22] Satilmis B., Alnajrani M.N., Budd P.M. (2015). Hydroxyalkylaminoalkylamide PIMs: Selective adsorption by ethanolamine-and diethanolamine-modified PIM-1. Macromolecules.

[23] Weng X., Baez J.E., Khiterer M., Hoe M.Y., Bao Z., Shea K.J. (2015). Chiral polymers of intrinsic microporosity: Selective membrane permeation of enantiomers. Angew. Chem. Int. Ed..

[24] Du N., Robertson G.P., Song J., Pinnau I., Guiver M.D. (2009). High-performance carboxylated polymers of intrinsic microporosity (PIMs) with tunable gas transport properties. Macromolecules.

[25] Du N., Dal-Cin M.M., Robertson G.P., Guiver M.D. (2012). Decarboxylation-induced cross-linking of polymers of intrinsic microporosity (PIMs) for membrane gas separation. Macromolecules.

[26] Yong W.F., Chung T.-S. (2015). Miscible blends of carboxylated polymers of intrinsic microporosity (cPIM-1) and Matrimid. Polymer.

[27] Yong W.F., Li F.Y., Chung T.S., Tong Y.W. (2014). Molecular interaction, gas transport properties and plasticization behavior of cPIM-1/Torlon blend membranes. J. Membr. Sci..

[28] Salehian P., Yong W.F., Chung T.-S. (2016). Development of high performance carboxylated PIM-1/P84 blend membranes for pervaporation dehydration of isopropanol and CO_2_/CH_4_ separation. J. Membr. Sci..

[29] Liao K.-S., Lai J.-Y., Chung T.-S. (2016). Metal ion modified PIM-1 and its application for propylene/propane separation. J. Membr. Sci..

[30] Zhao H., Xie Q., Ding X., Chen J., Hua M., Tan X., Zhang Y. (2016). High performance post-modified polymers of intrinsic microporosity (PIM-1) membranes based on multivalent metal ions for gas separation. J. Membr. Sci..

[31] Wind J.D., Staudt-Bickel C., Paul D.R., Koros W.J. (2002). The effects of crosslinking chemistry on CO_2_ plasticization of polyimide gas separation membranes. Ind. Eng. Chem. Res..

[32] Satilmis B., Budd P.M. (2014). Base-catalysed hydrolysis of PIM-1: Amide versus carboxylate formation. RSC Adv..

[33] Santoso B., Yanaranop P., Kang H., Leung I.K., Jin J. (2017). A Critical Update on the Synthesis of Carboxylated Polymers of Intrinsic Microporosity (C-PIMs). Macromolecules.

[34] Jeon J.W., Kim D.-G., Sohn E.-H., Yoo Y., Kim Y.S., Kim B.G., Lee J.-C. (2017). Highly Carboxylate-Functionalized Polymers of Intrinsic Microporosity for CO2-Selective Polymer Membranes. Macromolecules.

[35] McMurry J. (2008). Organic Chemistry.

[36] Yanaranop P., Santoso B., Etzion R., Jin J. (2016). Facile conversion of nitrile to amide on polymers of intrinsic microporosity (PIM-1). Polymer.

[37] Satilmis B., Budd P.M., Uyar T. (2017). Systematic hydrolysis of PIM-1 and electrospinning of hydrolyzed PIM-1 ultrafine fibers for an efficient removal of dye from water. React. Funct. Polym..

[38] Olah G.A., Olah J.A. (1965). Reactions of Amides and Sulfonamides with Nitrosonium Salts. J. Org. Chem..

[39] Bhole Y.S. (2007). Investigations on Gas Permeation and Related Physical Properties of structurally Architectured Aromatic Polymers (Polyphenylene Oxides and Polyarylates), Polyarlate-Clay Nanocomposites and Poly (Ionic Liquid). Ph.D. Thesis.

[40] Sakaguchi T., Yumoto K., Shiotsuki M., Sanda F., Yoshikawa M., Masuda T. (2005). Synthesis of poly (diphenylacetylene) membranes by desilylation of various precursor polymers and their properties. Macromolecules.

[41] Krasnokutskaya E.A., Semenischeva N.I., Filimonov V.D., Knochel P. (2007). A new, one-step, effective protocol for the iodination of aromatic and heterocyclic compounds via aprotic diazotization of amines. Synthesis.

[42] Filimonov V.D., Trusova M., Postnikov P., Krasnokutskaya E.A., Lee Y.M., Hwang H.Y., Kim H., Chi K.-W. (2008). Unusually stable, versatile, and pure arenediazonium tosylates: Their preparation, structures, and synthetic applicability. Org. Lett..

[43] Borikar S.P., Paul V. (2010). *N*-Nitrosation of Secondary Amines Using *p*-TSA-NaNO_2_ as a Novel Nitrosating Agent Under Mild Conditions. Synth. Commun..

[44] Søndergaard R.R., Norrman K., Krebs F.C. (2012). Low-temperature side-chain cleavage and decarboxylation of polythiophene esters by acid catalysis. J. Polym. Sci. Part A Polym. Chem..

[45] Philippides A., Budd P.M., Price C., Cuncliffe A.V. (1993). The nitration of polystyrene. Polymer.

